# Editorial: Systemic immune dysregulation in malignant disease: Insights, monitoring and therapeutic exploitation

**DOI:** 10.3389/fonc.2023.1182081

**Published:** 2023-04-03

**Authors:** Petros Christopoulos, Udo S. Gaipl

**Affiliations:** ^1^ Department of Oncology, Thoraxklinik and National Center for Tumor Diseases at Heidelberg University Hospital, Heidelberg, Germany; ^2^ Translational Lung Research Center at Heidelberg University Hospital, Member of the German Center for Lung Research (DZL), Heidelberg, Germany; ^3^ Translational Radiobiology, Universitätsklinikum Erlangen, Friedrich-Alexander-Universität Erlangen-Nürnberg, Erlangen, Germany; ^4^ Comprehensive Cancer Center Erlangen-European Metropolitan Area Nürnberg (CCC ER-EMN), Erlangen, Germany

**Keywords:** immune checkpoint inhibitors, immune microenviroment, immune dysregulation, cell therapies, biomarkers

Each tumor does not only trigger immune responses aiming to control its growth, but also causes profound immune dysregulation in the host: chronic antigen stimulation, contact-dependent effects through invariant receptors, and paracrine or systemic release of mediators induce alterations on virtually every immune cell type in the body. Precise characterization of these changes has key importance for the development of novel immunomodulatory strategies alone or in combination with standard modalities, such as radio- and chemotherapy (1, 2). Aim of this Research Topic was to provide a broad and comprehensive overview about recent discoveries in the field of cancer-associated systemic immune dysregulation, explore pathophysiologic links between the genetic contexture and immunologic tumor microenvironment (TME), analyze the potential prognostic and predictive value of immunologic biomarkers, and highlight opportunities for direct therapeutic exploitation using novel drugs or in the context of cell therapies.

A main subject was the potential clinical utility of blood-based and other biomarkers to guide patient management under immunotherapy (IO, **Table 1**). Using cytometric bead arrays for multiplex quantification of serum proteins, Schindler et al. demonstrated that blood cytokines can serve as predictors of efficacy and toxicity for PD-(L)1-treated non-small-cell lung cancer patients (NSCLC), but are not suitable for disease monitoring, since serial measurements cannot capture disease progression. This resembles the properties of simple blood-based biomarkers, like the neutrophil-to-lymphocyte ratio and advanced lung inflammation index (ALI) (17, 18), but is in contrast to circulating tumor DNA (ctDNA) assays, which have recently demonstrated superiority compared to radiologic imaging for the longitudinal monitoring of lung and other cancers under immunotherapy or targeted drugs (19, 20). Besides, Zhou et al. (4) demonstrated how machine learning methods can be leveraged in order to improve the predictive power of single blood-based biomarkers, like the C-reactive protein (CRP) and platelet-to-lymphocyte ratio (PLR), in order create complex, more accurate predictors for the immune-related adverse events (irAE) of cancer immunotherapy. Complementary immune cell-based matrices obtained from the peripheral blood of cancer patients might succeed to predict therapy responses more easily and accurately in the future (21).

**Table 1:** Articles published as part of the current research topic.

#	Journal	Title	Ref.
1	FO	Serum cytokines predict efficacy and toxicity, but are not useful for disease monitoring in lung cancer treated with PD–(L)1 inhibitors	(3)
2	FI	Elucidation of the application of blood test biomarkers to predict immune–related adverse events (irAEs) in atezolizumab–treated NSCLC patients by using machine learning methods	(4)
3	FO	The prominent role of the S100A8/S100A9–CD147 axis in the progression of penile cancer	(5)
4	FI	A New Thinking: Deciphering the Aberrance and Clinical Implication of IGF Axis Regulation Pattern in Clear Cell Renal Cell Carcinoma	(6)
5	FI	The HSP Immune Network in Cancer	(7)
6	FI	m6A modification patterns with distinct immunity, metabolism, and stemness characteristics in soft tissue sarcoma	(8)
7	FI	Immune Checkpoint Inhibitor–Induced Cerebral Pseudoprogression: Patterns and Categorization	(9)
8	FI	Human papillomavirus infection can alter the level of tumour stemness and T cell infiltration in patients with head and neck squamous cell carcinoma	(10)
9	FI	Phenotypic and functional analysis of γδ T cells in the pathogenesis of human T–cell lymphotropic virus type 1 infection	(11)
10	FI	Systemic immune dysregulation correlates with clinical features of early NSCLC	(12)
11	FI	Picturing of the lung tumor cellular composition by multispectral flow cytometry	(13)
12	FI	Characterization of the immune cell landscape in CRC: clinical implications of tumour–infiltrating leukocytes in early– and late–stage CRC	(14)
13	FI	Sarcoidosis following Hematopoietic Stem Cell Transplantation: clinical characteristics and HLA associations	(15)
14	FI	Utility and drawbacks of Chimeric Antigen Receptor T cell (CAR–T) therapy in lung cancer	(16)

FI, Frontiers in Immunology; FO, frontiers in Oncology.

Derangement of T-cell immunity also plays an essential role in the pathogenesis of cancer and treatment-related complications (**Figure 1**). One prominent example is cerebral pseudoprogression, which is caused by immune cell influx rather than tumor growth, may affect approximately 5% of patients receiving PD-(L)1 inhibitors, and requires meticulous radiologic criteria for accurate diagnosis in order to avoid therapeutic mistakes, as Urban et al. demonstrated. Alterations of cellular immunity are particularly prominent in case of tumors caused by chronic infection. This was well illustrated by the study of Meng et al. who analyzed head-and-neck squamous cell carcinoma (HNSCC) and revealed a lower degree of cancer cell stemness, heavier CD8^+^ cell infiltration, stronger expression of immune checkpoint molecules of T-cells, and elevated CCL4 in HPV-positive compared to HPV-negative tumor cells, as assessed by bulk and single-cell RNAseq data from various patient cohorts and cell lines. These data provide a mechanistic basis for the higher IO-sensitivity and more favorable prognosis of HPV-positive compared to HPV-negative HNSCC. Similar observations were reported by Huang et al. in soft-tissue sarcoma (STS), where a lower degree of stemness, more pronounced immune cell infiltrates, better sensitivity to chemotherapy and immunotherapy, as well as longer survival were observed in tumors with less N6-methyladenosine (m6A) RNA methylation. Besides, γδ T cells are important for the control of another virally induced pathology, *i.e.* HTLV-1-associated adult T-cell leukemia/lymphoma (ATLL) and tropical spastic paraparesis (TSP), as highlighted by Ruggieri et al.: cytotoxic Vγ9δ2 lymphocytes can eliminate eukaryotic cells expressing the HTLV-1 proteins HBZ or Tax, while progression from asymptomatic HTLV-1 infection to clinically overt ATLL/TSP is accompanied by depletion of the protective effectors *in vivo*. Instrumental for these insights was fine-granular analysis of T-cell receptor (TCR) repertoire in HTLV-1 infected patients and healthy donors using a custom spectratyping protocol that could differentiate between very similar transcripts belonging to various human Vγ and Vδ families (22). This is proof-of-principle for the potential clinical utility of comprehensive TCR profiling to elucidate pathogenesis and refine patient stratification in various cancers, as also suggested by earlier pivotal studies in melanoma, large-cell neuroendocrine lung carcinoma and indolent B-cell lymphoma (B-NHL) (23–26).

**Figure 1:**
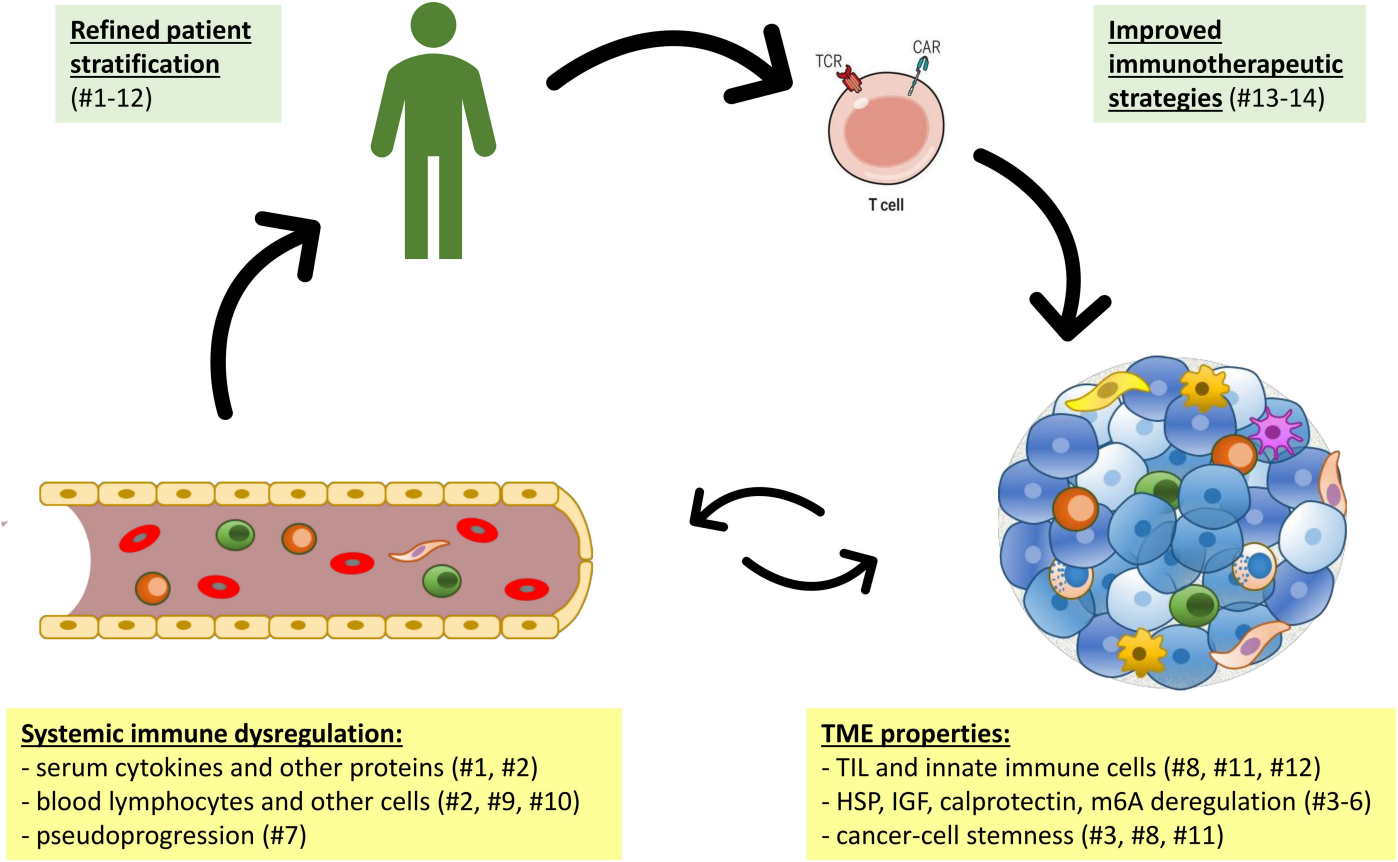
The interplay of systemic immune dysregulation and the tumor microenvironment (TME) of cancer as basis for clinical exploitation. Articles of the current research topic are shown in parentheses numbered according to **Table 1**.

Another important principle nicely demonstrated by the study of Ruggieri et al. is that systemic immune dysregulation and lymphocyte aberrations worsen with further disease progression in advanced cancer. Similar findings have also been described in many other hematologic and solid tumors, for example B-NHL and NSCLC Hao et al. (27, 28). Therefore, studies focusing on early–stage disease are of key importance to deconvolute the complex pathogenetic chains. Using multiparametric flow cytometry to analyze longitudinal blood samples from NSCLC patients, Hao et al. could demonstrate that significant depletion of multiple T–cell subsets alongside systemic immune activation are present already in patients with localized disease and aggravated after radical surgery, for example CD4^+^ cell counts and the CD4/CD8–cell ratio significantly decreased, but NKT increased in many patients after tumor resection. Thus, quantification of peripheral immune cell subsets could be used alongside TCR–based and ctDNA–based liquid biopsies in the future to improve monitoring of lung and other cancers (29, 30), as well as guide application of consolidative immunotherapies (31, 32). These tools could pave the way for personalized strategies of perioperative immunotherapy, which will become a main objective of translational research in the next few years, after the recent approval of the first adjuvant and neoadjuvant PD–(L)1 inhibitor treatments in thoracic oncology based on the Impower010 and Checkmate–816 trials, respectively (33, 34).

Accumulating evidence suggests that alterations of blood lymphocytes in lung and other cancers originate in the TME, which is hijacked by the growing tumor and transformed in an immunosuppressive niche (1). For example, using multispectral flow cytometry with hierarchical clustering to analyze samples from diverse murine lung cancer models, Olesch et al. could demonstrate alterations of CD324^+^ epithelial cells, alveolar macrophages, dendritic cells (DCs) and endothelial cells in animals with primary tumors, while fibroblasts, vascular smooth muscle cells, monocytes (Ly6C^+^ and Ly6C^–^) and neutrophils were elevated in metastatic models. On the other hand, Bazzi et al. could identify alterations of mast cells and DCs as predominant prognostic markers in early colorectal cancer (CRC) by comparative CIBERSORT analysis *vs.* the adjacent normal tissue. These changes are facilitated by deregulation of several important biological axes in human cancers, whose precise characterization could reveal specific therapeutic vulnerabilities. For example, expression of the calprotectin–receptor CD147 on penile cancer cells was associated with increased counts of S100A8^+^/S100A9^+^ neutrophil–derived suppressor cells in the TME and an elevated risk of metastasis (Mohr et al.). Furthermore, lower expression of IGF regulators in renal cell cancer (RCC) was linked to reduced infiltration by CD8^+^, Th1, and plasmacytoid DCs, activation of multiple metabolic pathways that fuel cancer progression, and lower sensitivity to antiangiogenics and immunotherapy, despite a higher tumor mutational burden (Jiang et al.). A third example are heat–shock proteins (HSP), which support tumor development by both intracellular and paracrine effects by regulating a wide array of biological processes, including unfolded protein responses, mitochondrial bioenergetics, apoptosis, autophagy, necroptosis, lipid metabolism, angiogenesis, cancer cell stemness, epithelial–mesenchymal transition and tumor immunity (Albakova and Mangasarova). At the same time, they can also serve as danger signal, for example by delivering antigens to DCs, or by direct activation of NK cells (35). The predictive value of serum Hsp70 is currently under investigation (36). Many recent insights into TME processes have relied on global transcriptomic profiling of tumor biopsies to dissect the TME at the functional level, but this method is still too resource–intensive for routine application. An attractive alternative is Nanostring–based targeted RNA profiling, a hybridization method suitable for formalin–fixed paraffin–embedded (FFPE) tissue specimens that requires very small amounts of input material, can be incorporated into the routine molecular workup, and may provide useful prognostic information for newly diagnosed with lung and other cancers (37–40). On the other hand, purely genetic markers, like the tumor mutational burden (TMB), pose technical challenges and have failed to meet expectations (41). Considering the significant variability among patients and cancer entities, wide adoption of practicable TME analysis methods will be crucial for personalized insights and tailored immunotherapeutic approaches. That being said, there are also important similarities across tumors, like the blood lymphopenia, which is a common feature and key adverse prognostic factor of carcinomas, sarcomas, and lymphomas (42). The sole exception to this rule appear to be thymic epithelial tumors, which uniquely cause an accumulation of hyporesponsive CD247–deficient naive T–cells in the periphery due to the unique thymic role in T–cell maturation (43, 44).

From a clinical standpoint, of utmost importance is the therapeutic exploitation of tumor–related immunologic changes. Up until a few years ago, the most effective immunotherapy for malignant disease has been allogeneic hematopoietic stem cell transplantation (HSCT), which represented the only curative option for patients with refractory hematologic cancers, like relapsed acute myeloid leukemia (AML) (45). However, this therapy was notoriously toxic, as the preceding conditioning regimens and subsequent graft–versus–host disease (GvHD) caused significant morbidity and mortality. One rare and exceptionally mild complication is secondary sarcoidosis, which occurs with a very low frequency <1% following HSCT, particularly in the presence of specific HLA–haplotypes, and usually resolves under standard glucocorticoid treatment without long–term sequelae, as observed by Wurm-Kuczera et al.. A newer cell–based cancer therapeutic are chimeric antigen receptor (CAR)–T cells, which have revolutionized the treatment of hematologic malignancies, but face two major challenges in application against lung and other solid tumors: the paucity of suitable dispensable extracellular target antigens, and an immunosuppressive TME that hinders penetration and activation of effector cells, as described by Kandra et al.. Very promising in this regard is the development of transgenic TCR–T cell therapies, which can be directed against the much larger pool of intracellular antigens and further augmented by genetic engineering in order to “heat–up” cold tumors for increased efficacy (46). Another innovative strategy with huge momentum currently are multi–specific antibodies, which can recruit endogenous T– or NK–cells against tumor cells bearing specific surface or even intracellular antigens (47). Important advantages of antibody– *vs.* cell–based strategies are immediate, off–the–shelf availability and better tolerability, since no previous conditioning is needed, so that these therapies can be started faster and combined with any other modality, like radiotherapy, chemotherapy or other immunotherapies for synergistic effects (48–50).

Since the advent of PD–(L)1 inhibitors, modern cancer medicine has been increasingly focused on the better unraveling, monitoring and reversal of the cancer–associated immune dysregulation for further therapeutic progress. Recent developments in these fields offer a justified hope for cure of several cancers in the near future, a snapshot of which the current Research Topic aspired to capture.

## Author contributions

Both authors made a substantial, direct, and intellectual contribution to the work and approved it for publication.
